# Subcutaneous Administration of Bortezomib: Strategies to Reduce Injection Site Reactions

**DOI:** 10.6004/jadpro.2012.3.6.8

**Published:** 2012-11-01

**Authors:** Sandra Kurtin, Carol S. Knop, Todd Milliron

**Affiliations:** From University of Arizona Cancer Center, Tucson, Arizona; Northwestern Medical Faculty Foundation, Chicago, Illinois; and University of Maryland Medical System, Baltimore, Maryland

## Abstract

Bortezomib (Velcade) is approved by the FDA for IV or SC injection in select patients with multiple myeloma or mantle cell lymphoma. The SC route functions as an alternative to IV administration for patients with poor IV access. Learn about effective strategies used to reduce injection site reactions that can occur with SC delivery.

Bortezomib (Velcade), a reversible proteasome inhibitor, is approved by the US Food and Drug Administration (FDA) for intravenous (IV) or subcutaneous (SC) injection in patients with multiple myeloma or those with mantle cell lymphoma who have received at least one prior therapy (Millennium Pharmaceuticals, Inc., 2012). The approval for myeloma was based on a phase III international randomized noninferiority trial evaluating the safety and efficacy of bortezomib administered either IV or SC using established doses (1.3 mg/m^2^ on days 1, 4, 8, and 11) in the treatment of patients with relapsed multiple myeloma (Moreau et al., 2011).

This key trial established the safety and efficacy of SC bortezomib following four cycles of therapy as being equivalent to IV bortezomib (Table 1). In addition, the incidence of peripheral neuropathy (PN), a common dose-limiting toxicity for bortezomib, was reduced with SC administration compared to the IV route: 38% vs. 58%, all grades; 6% vs. 16% for grade 3 or higher (Moreau et al., 2011). It is important to note, however, that the reconstitution of SC bortezomib is different from that for the IV administration (Millennium Pharmaceuticals, Inc., 2012).

**Table 1:**
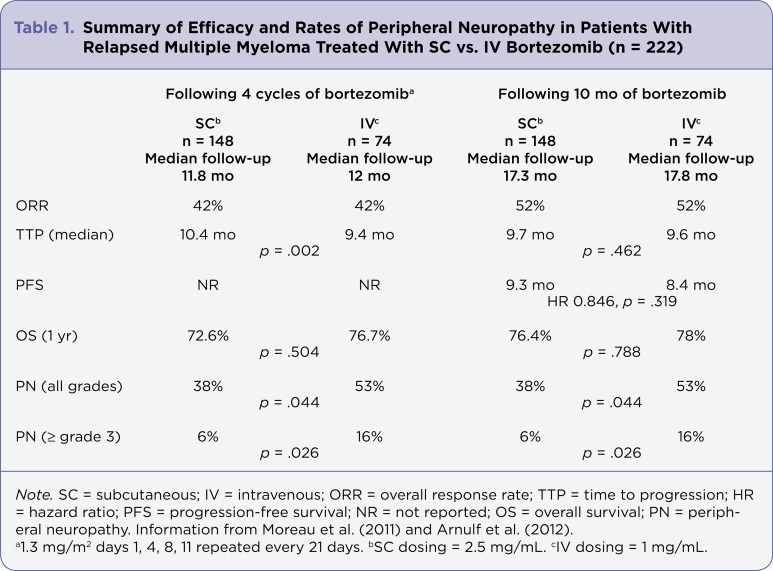
Table 1. Summary of Efficacy and Rates of Peripheral Neuropathy in Patients With Relapsed Multiple Myeloma Treated With SC vs. IV Bortezomib (n = 222)

These data have recently been updated, confirming sustained efficacy and safety at 10 months of follow-up in the same study population; see Table 1 (Arnulf et al., 2012). The incidence of PN has been shown to be reduced further in regimens using the weekly dosing schedule for bortezomib either as a single agent or in combination with other agents (Bringhen et al., 2010). No other significant changes in the adverse event profile for bortezomib were noted in the updated analysis (Arnulf et al., 2012).

## Subcutaneous Drug Administration

The SC route offers the ability to deliver medication into the adipose tissue with more rapid absorption than oral administration and a longer duration of drug activity than intramuscular administration, and it functions as an alternative to IV administration for patients with poor IV access. To be effective, medications administered using the SC route must be delivered into the subcutaneous adipose tissue and must demonstrate consistent pharmacokinetics.

The most common medication delivered through the SC route is insulin, thus its administration techniques have been adopted from that clinical experience. Two recent literature reviews evaluating SC injection technique for the administration of insulin confirm limited evidence-based guidelines (Annersten & Willman, 2005; Frid et al., 2010). Many of the papers included in these reviews focused on patient self-injection techniques in adolescents and children. The key recommendations offered in these reviews include the continuation of accepted techniques for site preparation using an antiseptic agent and rotation of SC injection sites (Annersten & Willman, 2005; Frid et al., 2010).

Key recommendations to ensure delivery of the medication into adipose tissue and not muscle include site selection based on skin thickness and subcutaneous fat, appropriate skin fold technique prior to injection, injection angle, and needle length. The thickness of the skin in adults is between 1.9 and 2.4 mm, with little variation in different body locations or among individuals of varying race, sex, or body mass index (Gibney, Arce, Byron, & Hirsch, 2010). There are, however, differences in the thickness of subcutaneous tissue by site and sex, with women having greater SC thickness than men, SC thickness being greatest in the abdomen, and SC thickness being reduced in lean individuals (Gibney et al., 2010; Vora, Peters, Burch & Owens, 1992; see Table 2).

**Table 2:**
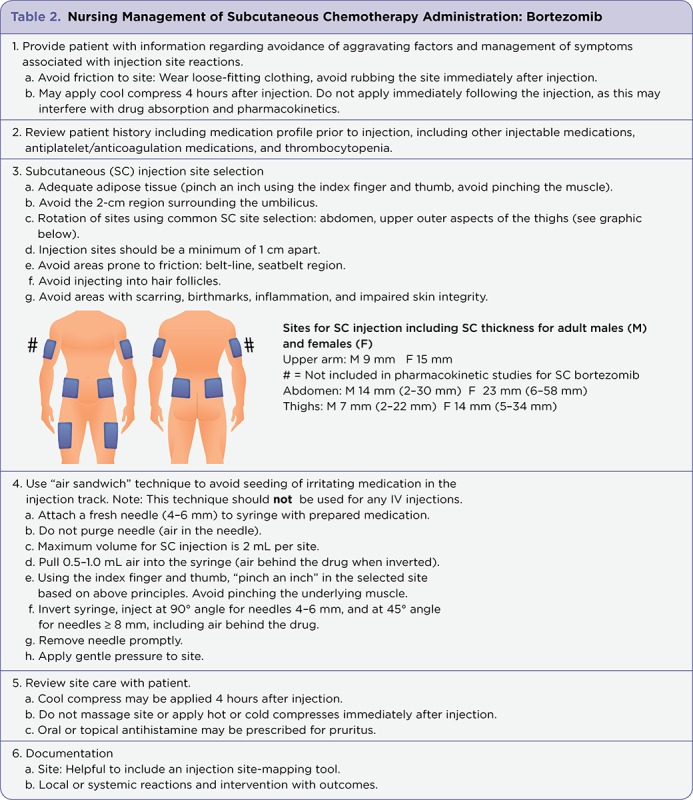
Table 2. Nursing Management of Subcutaneous Chemotherapy Administration: Bortezomib

Commonly available needle sizes for insulin administration are 4 mm (5/32"), 5 mm (3/16"), 8 mm (5/16"), and 12.7 mm (1/2"). Needles that are between 4 and 6 mm have been proven effective in adults of all body sizes, allowing penetration of the skin into the adipose tissue and avoiding intramuscular penetration (Gibney et al., 2010; Vora et al., 1992). Subcutaneous injections using these needles should be given at a 90° angle. Subcutaneous injections given using needles > 6 mm should be administered using a 45° angle to avoid intramuscular penetration, which is associated with different pharmacokinetics.

## Injection Site Reactions

Unlike insulin, which is self-administered by patients, the majority of chemotherapeutic agents are administered by registered nurses. Injection site reactions have been reported in clinical trials and postmarketing experiences for chemotherapeutic agents administered using the SC route (Kurtin & Demakos, 2010; Murray et al., 2012). Although injection site reactions are rarely severe, they may be associated with discomfort, body image concerns due to visible skin changes, and emotional distress for the patient. Patients experiencing injection site reactions may be reluctant to continue treatment using the SC route in the presence of such reactions (Saunders, Caon, Smrtka, & Shoemaker, 2010). Experiences gained from the SC administration of 5-azacitidine, a hypomethylating agent used to treat myelodysplastic syndromes, have provided insight into strategies to reduce local injection site reactions in patients receiving SC chemotherapeutic agents. Use of the "air sandwich" technique has been effective in limiting the severity of injection site reactions during azacitidine administration (Kurtin & Demakos, 2010; Murray et al., 2012); see Table 2 for details.

**Figure 1:**
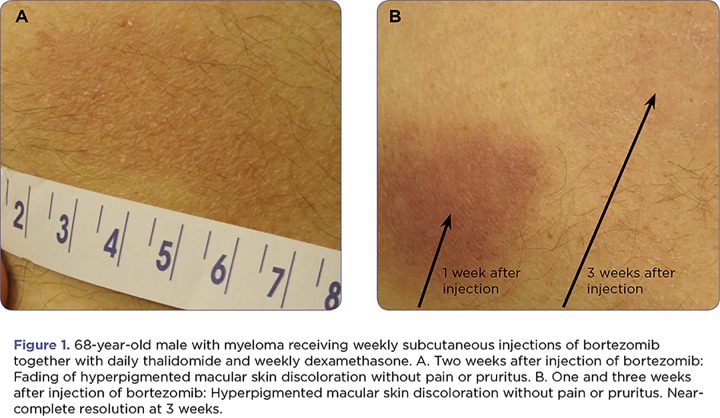
Figure 1. 68-year-old male with myeloma receiving weekly subcutaneous injections of bortezomib together with daily thalidomide and weekly dexamethasone. A. Two weeks after injection of bortezomib: Fading of hyperpigmented macular skin discoloration without pain or pruritus. B. One and three weeks after injection of bortezomib: Hyperpigmented macular skin discoloration without pain or pruritus. Near-complete resolution at 3 weeks.

**Figure 2:**
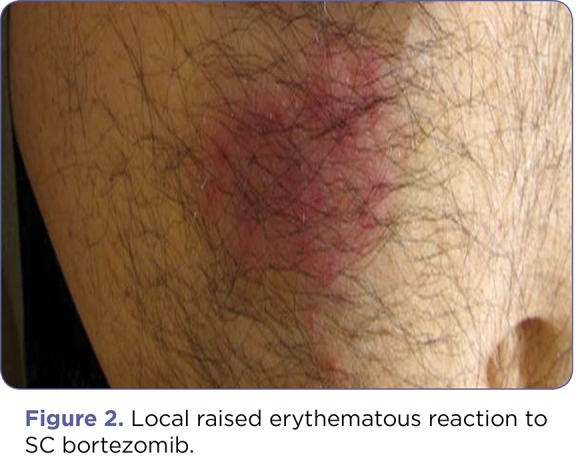
Figure 2. Local raised erythematous reaction to SC bortezomib.

**Figure 3:**
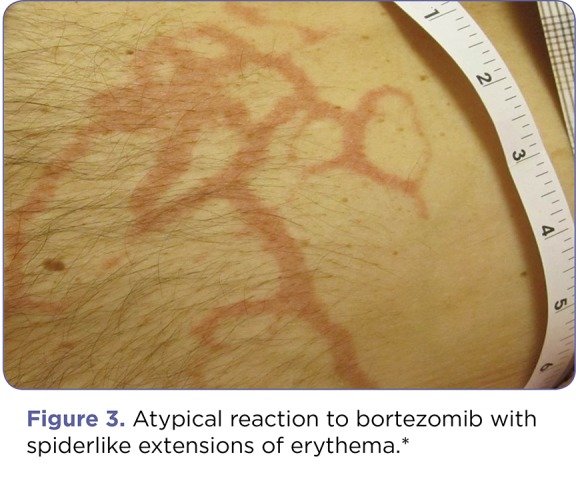
Figure 3. Atypical reaction to bortezomib with spiderlike extensions of erythema. Complete resolution occurred within 48 hours.

Injection site reactions are common but generally mild with SC administration of bortezomib. The most common finding is a 2-cm to 3-cm area of hyperpigmentation at the injection site with or without pruritus, which is self-limiting and resolves over a period of days to weeks (Figure 1). More severe reactions have been reported in clinical practice, including areas of hyperpigmentation exceeding 10 cm, variable degrees of induration, and in some cases scab formation at the injection site (Figures 2 and 3). Treatment for injection site reactions may include the administration of oral antihistamines. Topical application of cool compresses, antihistamines, or corticosteroids is not recommended within 4 hours of drug administration, to avoid any changes in pharmacokinetics. After 4 hours, any topical treatment should be applied gently to avoid unnecessary friction to the site.

## Developing Needed Protocols

Clinical trials using the SC route for other chemotherapeutic agents are in process. One agent administered via the SC route, omacetaxine mepesuccinate (Synribo), was approved by the FDA on October 26, 2012, for the treatment of adult patients with chronic or accelerated phase Philadelphia-positive chronic myeloid leukemia with resistance and/or intolerance to two or more tyrosine kinase inhibitors. Adopting a protocol for SC administration of chemotherapeutic agents that reduces the incidence and severity of injection site reactions, while ensuring delivery of the drug into the adipose tissue, will facilitate continuation of treatment using the SC route and its associated benefits: treatment efficacy, a reduced incidence of PN in the case of bortezomib, and limited time spent by patients in the clinical setting. Proper injection site protocols should include guidelines for site selection, needle size, volume of medication per site, patient education for postinjection care, and guidelines for proper documentation.
